# miR‐34a^−/−^ mice are susceptible to diet‐induced obesity

**DOI:** 10.1002/oby.21561

**Published:** 2016-07-05

**Authors:** Christopher A. Lavery, Mariola Kurowska‐Stolarska, William M. Holmes, Iona Donnelly, Muriel Caslake, Andrew Collier, Andrew H. Baker, Ashley M. Miller

**Affiliations:** ^1^ Institute of Cardiovascular & Medical Sciences, College of Medical, Veterinary and Life Sciences, University of GlasgowGlasgowUK; ^2^ Institute of Infection, Immunity and Inflammation, College of Medical, Veterinary and Life Sciences, University of GlasgowGlasgowUK; ^3^ Glasgow Experimental MRI Centre, Institute of Neuroscience and Psychology, University of GlasgowGlasgowUK; ^4^ Ayr Hospital, National Health Service: Ayrshire & ArranAyrUK

## Abstract

**Objective:**

MicroRNA (miR)−34a regulates inflammatory pathways, and increased transcripts have been observed in serum and subcutaneous adipose of subjects who have obesity and type 2 diabetes. Therefore, the role of miR‐34a in adipose tissue inflammation and lipid metabolism in murine diet‐induced obesity was investigated.

**Methods:**

Wild‐type (WT) and miR‐34a^−/−^ mice were fed chow or high‐fat diet (HFD) for 24 weeks. WT and miR‐34a^−/−^ bone marrow‐derived macrophages were cultured *in vitro* with macrophage colony‐stimulating factor (M‐CSF). Brown and white preadipocytes were cultured from the stromal vascular fraction (SVF) of intrascapular brown and epididymal white adipose tissue (eWAT), with rosiglitazone.

**Results:**

HFD‐fed miR‐34a^−/−^ mice were significantly heavier with a greater increase in eWAT weight than WT. miR‐34a^−/−^ eWAT had a smaller adipocyte area, which significantly increased with HFD. miR‐34a^−/−^ eWAT showed basal increases in *Cd36*, *Hmgcr*, *Lxrα*, *Pgc1α*, and *Fasn*. miR‐34a^−/−^ intrascapular brown adipose tissue had basal reductions in *c/ebpα* and *c/ebpβ*, with *in vitro* miR‐34a^−/−^ white adipocytes showing increased lipid content. An F4/80^high^ macrophage population was present in HFD miR‐34a^−/−^ eWAT, with increased *IL‐10* transcripts and serum IL‐5 protein. Finally, miR‐34a^−/−^ bone marrow‐derived macrophages showed an ablated CXCL1 response to tumor necrosis factor‐α.

**Conclusions:**

These findings suggest a multifactorial role of miR‐34a in controlling susceptibility to obesity, by regulating inflammatory and metabolic pathways.

## Introduction

The growing incidence of obesity worldwide has presented new therapeutic challenges, making it critical to understand the underlying mechanisms of obesity‐associated pathology. In 2014, the World Health Organization estimated that 12.9% of adults have obesity (BMI ≥30 kg/m^2^, age ≥18 years) worldwide, with the Americas (26.8%) and Europe (23%) showing the highest percentage. Obesity is associated with increased low‐grade, chronic inflammation that predisposes individuals to a number of comorbidities, e.g., type 2 diabetes (T2D), dyslipidemia, cardiovascular diseases (CVDs), and cancers 1, 2.

The main proponent of weight gain during obesity is white adipose tissue (WAT). However, far from being just a lipid storage vessel, WAT can modulate the inflammatory response through secretion of inflammatory cytokines, including CCL2, CXCL8, tumor necrosis factor (TNF)‐α, interleukin (IL)−1β, and IL‐6 1, 2, 3. In opposition, brown adipose tissue (BAT) expends fatty acids (FA) to produce heat through mitochondrial action and uncoupled protein 1 (UCP1), maintaining body temperature. Peroxisome proliferator‐activated receptor (PPAR)‐γ coactivator (PGC)−1α is important for BAT adaptive thermogenesis, increasing mitochondrial biogenesis and UCP1 expression and promoting brown adipocyte differentiation 4.

MicroRNAs (miRNAs/miRs) are small (19–25 nt), noncoding RNAs, which primarily bind the 3′ untranslated region of target mRNA, mediated by Argonaute proteins within the cytoplasmic RNA‐inducing silencing complex (RISC). Before RISC loading, miRNAs form a duplex consisting of a mature (5p) and star (3p) strand, but usually only the 5p is loaded. mRNA is silenced through inhibition of translation or degradation 5.

The miR‐34 family consists of 34a, 34b, and 34c, with miR‐34a encoded on chromosome 1p36.22 and miR‐34b and c on chromosome 11q23.1 6. Interestingly, miR‐34a appears to be dysregulated during obesity, with increased serum miR‐34a transcripts found in patients who have excess weight (BMI ≥25 kg/m^2^) with T2D, compared with patients who have excess weight without diabetes 7. In addition, studies have shown a correlation with increasing BMI and miR‐34a transcripts in human subcutaneous WAT (scWAT) and increased miR‐34a transcripts over subcutaneous adipocyte differentiation *in vitro* 8, 9. Further to its pro‐diabetes role, miR‐34a transcripts have been positively associated with destruction of primary β‐islet cells and cell lines, upon inflammatory stimulation, with decreased insulin secretion, through inhibition of VAMP2 10, 11. Inflammatory roles of miR‐34a have been proposed, with lipopolysaccharide‐induced inflammation reducing miR‐34a expression in macrophage cell lines, and miR‐34a mimics reducing IL‐6 and TNF‐α expression 12. Furthermore, miR‐34a has been shown to target the soluble IL‐6 receptor 13.

Adipose tissue macrophages (ATMs) represent a major immune infiltrate into the obese WAT (predominantly visceral), increasing from around 12% to 41% 14. Increasing inflammatory cytokines, particularly CCL2, recruits monocytes into the WAT that differentiate into ATMs 15. A high percentage are proinflammatory F4/80^+^ CD11b^+^ CD11c^+^ M1 ATMs in high‐fat diet (HFD)‐fed rodents, and depletion of these cells has shown a reversal in inflammation and insulin resistance 16. Other immune cells, such as neutrophils, have also been shown to contribute to insulin resistance and obesity‐induced inflammation 17.

Therefore, the aims of this study were to examine the metabolic and inflammatory role of miR‐34a in a murine, diet‐induced obesity model, using miR‐34a^−/−^ mice; specifically, examining miR‐34a's role in adipocytes and macrophages during obesity. Our findings show that miR‐34a^−/−^ mice are susceptible to weight gain, likely through basal metabolic gene changes in adipose caused by dysregulation of PGC‐1α.

## Methods

### Murine studies

All *in vivo* studies used 7‐week‐old, male, wild‐type (WT) or miR‐34a^−/−^ (B6.Cg‐Mir34a^tm1Lhe^/J) C57BL/6 mice (Jackson Labs, Sacramento, CA) bred in‐house. Mice were fed normal chow (chow) or HFD (0.15% cholesterol and 21% lard: Special Diet Services, Essex, UK) *ad libitum* for 24 weeks. Weekly weights, monthly fasting blood glucose, and glucose tolerance test (GTT) measurements were recorded. Fasting (16–18 h) blood glucose measurements were recorded using an Accu‐Chek® Mobile glucose monitor (Roche, Burgess Hill, UK: Model U1; Max: 33.33 mmol/L). GTT measurements were taken every 30 min, following I.P. administration of 1 g/kg glucose, in 0.9% saline solution. All studies were carried out in a registered research facility, under UK Home Office guidelines.

### Human studies

Omental adipose tissue biopsies were collected from six patients with obesity (five women and one man), T2D (HbA1c = 7.5 ± 0.97%), and/or sleep apnea, undergoing bariatric surgery. Patients were on metformin (BMI: 42.13 ± 3.24; age: 50.17 ± 8.08 years). All patients gave written informed consent before inclusion in the study, with approval from the West of Scotland Research Ethics Service (REC Ref 12/WS/0158).

Additional methods can be found in Supporting Information.

## Results

### Expression of miR‐34a in obese adipose tissue and *in vitro* bone marrow‐derived macrophages

**Figure 1:**
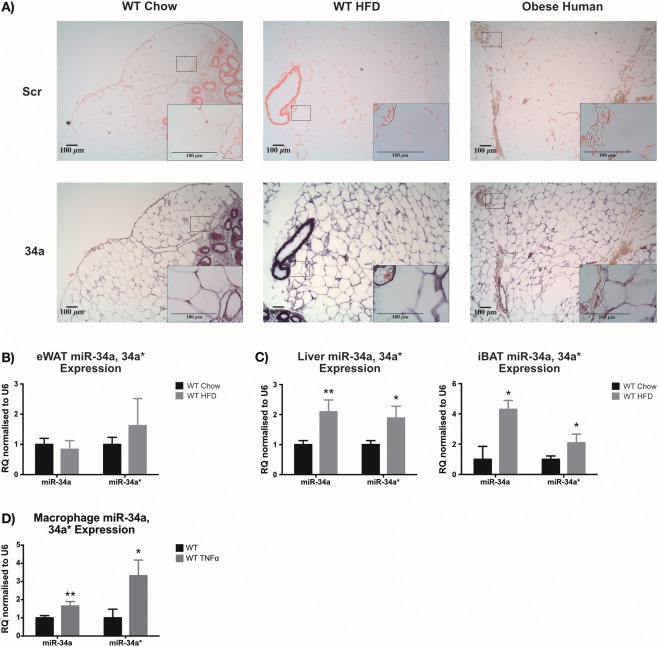
Expression of miR‐34a in adipose tissue. (**A**) *In situ* hybridization (ISH) images of miR‐34a expression (purple) compared with a Scramble (Scr; red) negative control in epididymal (e)WAT from WT mice fed chow or high‐fat diet (HFD) for 24 weeks and omental adipose from patients with obesity undergoing bariatric surgery. RT‐qPCR data showing expression of miR‐34a and 34a* in (**B**) eWAT and (**C**) liver and intrascapular (i)BAT collected from WT mice 24 weeks after commencing a chow vs. HFD. (**D**) RT‐qPCR quantification of miR‐34a and 34a* transcripts in WT *in vitro* bone marrow‐derived macrophages (BMDM) ± 45.45 ng/mL tumor necrosis factor (TNF)‐α for 24 h. Data represented as relative quantification (RQ) with RQ_min_ − RQ_max_ values, normalized to RNU6; *n* = 3–4 for eWAT, *n* = 5–6 for liver, *n =* 6 for iBAT, and *n =* 3 for BMDMs. *P < 0.05, **P < 0.01, unpaired Student's *t*‐test.

*In situ* hybridization showed miR‐34a distribution within visceral WAT was ubiquitous throughout the epididymal (e)WAT of WT mice on chow and HFD and omental adipose from bariatric surgery patients with obesity (Figure 1A). Similarly, no change was observed in miR‐34a or miR‐34a* transcripts between WT chow and HFD groups (Figure 1B). However, we observed increases in both miR‐34a and 34a* transcripts within the liver (*P =* 0.0088, *P =* 0.0247) and intrascapular (i)BAT (*P =* 0.0433, *P =* 0.0399) during HFD feeding (Figure 1C). Furthermore, we observed an increase in miR‐34a (*P =* 0.0086) and miR‐34a* (*P =* 0.0461) transcripts in WT bone marrow‐derived macrophages (BMDMs) when stimulated with TNF‐α (Figure 1D). We examined the other miR‐34 family members but found no expression changes (Supporting Information Figure S1).

### Endogenous miR‐34a regulated body weight and adipocyte size

**Figure 2:**
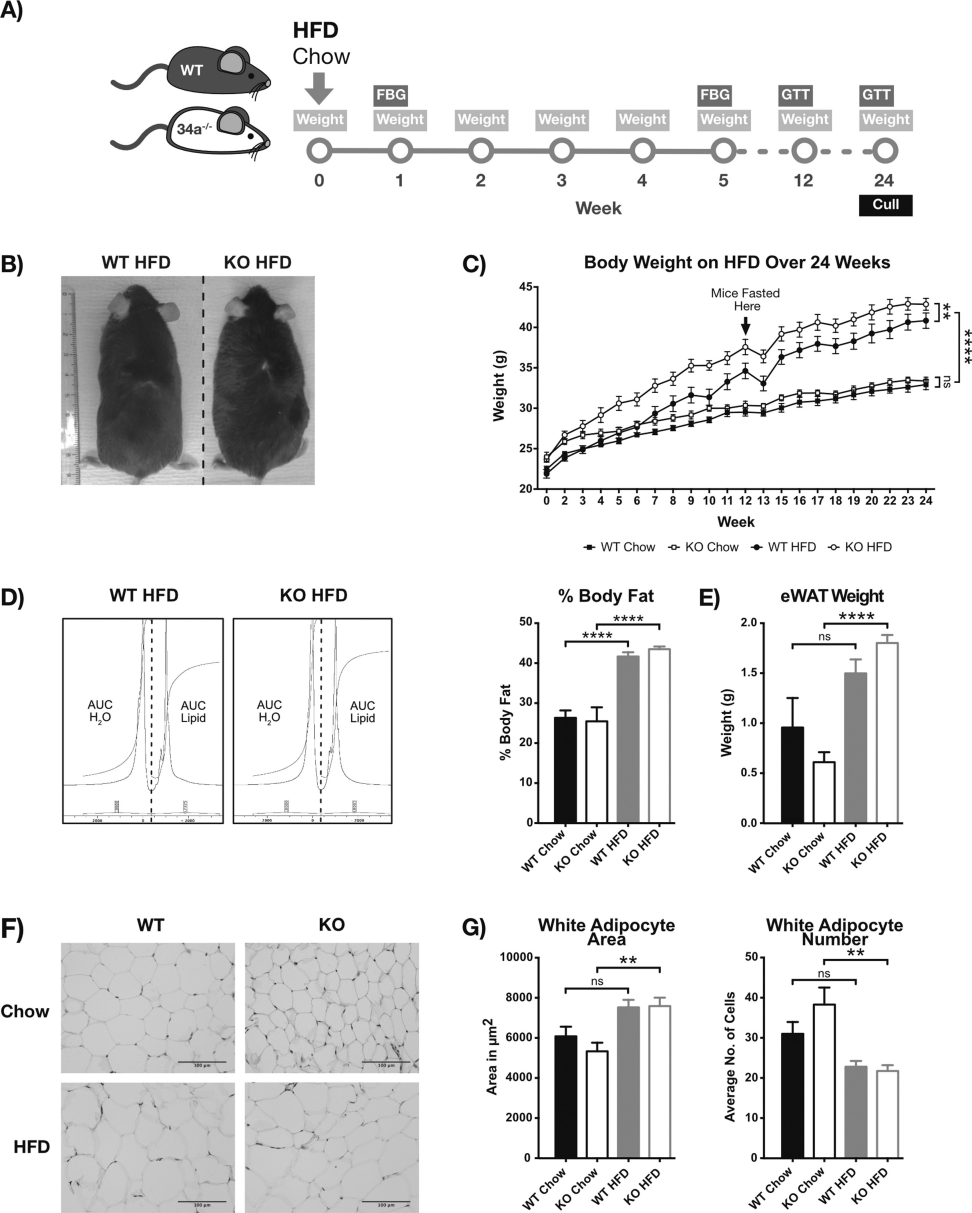
miR‐34a^−/−^ mice are susceptible to diet‐induced obesity. (**A**) Diagrammatic representation of the 24‐week *in vivo* study structure: high‐fat diet (HFD), fasting blood glucose (FBG), and glucose tolerance test (GTT). Data in panels **B**–**F** are from WT and miR‐34a^−/−^ (KO) mice on chow vs. HFD over 24 weeks of diet. (**B**) Representative image of HFD‐fed WT and KO mice at week 24 of study. (**C**) Body weight measurements over 24 weeks; *n =* 10 for KO chow and WT HFD and *n =* 9 for WT chow and KO HFD groups. (**D**) MRS analysis of mouse percentage body fat at week 24, calculated from integration ratios for lipid (AUC_lipid_) to water (AUC_H2O_) peaks; *n =* 6 for WT chow and HFD, *n =* 4 for KO HFD, and *n =* 3 for chow HFD groups. (**E**) Weight of excised epididymal (e)WAT at week 24 of study; *n =* 10 for KO chow and WT HFD and *n =* 9 for WT chow and KO HFD groups. (**F**) Representative H&E staining of eWAT excised in panel E. (**G**) Quantification of adipocyte area and number in panel F, with averages taken from ≥5 fields per section of *n =* 10 for KO chow and WT HFD and *n =* 8 for WT chow and KO HFD groups. Area measurements were converted from pixels to square centimeters using the image scale. All graphs represent mean values with SEM. **P < 0.01, ****P < 0.0001, one‐way ANOVA, with Bonferroni's multiple comparisons post‐test.

To investigate the role of endogenous miR‐34a in regulating obesity, we measured the weight and metabolic parameters of WT and miR‐34^−/−^ mice over 24 weeks on chow or HFD (Figure 2A). Before the study began, it was noted that miR‐34^−/−^ mice were heavier at week 0 (chow: 6.71%; *P =* 0.1311 and HFD: 8.07%; *P =* 0.0377) (Supporting Information Figure S2A). This was maintained in the chow group at week 2 (6.11%; *P =* 0.0312) and 3 (6.86%; *P =* 0.0798) but increased further in HFD‐fed miR‐34^−/−^ mice at week 2 (12%; *P <* 0.0001) and 3 (11.60%; *P =* 0.0009). HFD‐fed miR‐34a^−/−^ mice were significantly heavier (9.22 ± 0.56%; *P =* 0.0075) than WT counterparts over 24 weeks, by AUC analysis (Figure 2B, C). Body weights of chow‐fed miR‐34a^−/−^ mice were similar to WT controls. Interestingly, when we examined the total body fat percentage (AUC_lipid_) by MRS, both HFD‐fed WT and miR‐34a^−/−^ mice showed similar fat content (41.7 ± 1.04% vs. 43.5 ± 0.68%) (Figure 2D). These weight differences were not explained by food intake during metabolic cage studies (Supporting Information Figure S2B) or fasting serum leptin levels (Supporting Information Figure S2C).

However, *ex vivo* investigations revealed a significant increase in eWAT weight in HFD‐fed miR‐34a^−/−^ mice (0.61 ± 0.10 g to 1.80 ± 0.08 g; *P <* 0.0001) not observed in WT controls (Figure 2e). Liver weight remained unchanged (Supporting Information Figure S2D). Histological examination of the eWAT revealed smaller, more densely packed adipocytes within the miR‐34a^−/−^ chow tissues, compared with WT (Figure 2F, G). When stressed with a HFD, the eWAT from miR‐34a^−/−^ mice showed a significant increase in cell area (*P =* 0.0030) and decrease in cell number per field (*P =* 0.0013) not seen in WT, bringing them to WT levels.

To examine alterations in glucose homeostasis, we measured monthly fasting blood glucose and performed a GTT at week 24. However, we did not observe any changes between HFD‐fed WT and miR‐34a^−/−^ mice, both showing hyperglycemia from week 12 (*P <* 0.001) (Supporting Information Figure S2E) and similar glucose handling (Supporting Information Figure S2F). This was further compounded by no change in fasting serum insulin or resistin between HFD groups at week 24 (Supporting Information Figure S2F). Additionally, there was no change in fasting serum cholesterol and triglyceride measurements between WT and miR‐34a^−/−^ groups on either chow or HFD (Supporting Information Table S1).

### miR‐34a^−/−^ mice showed basal metabolic gene changes in eWAT and iBAT on chow diet

**Figure 3:**
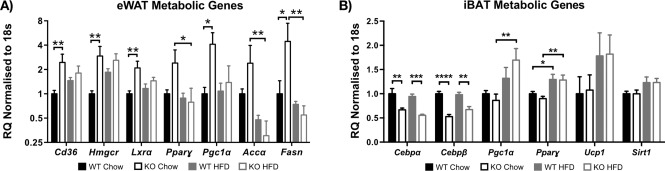
Altered expression of metabolic genes in epididymal (e)WAT and intrascapular (i)BAT of miR‐34a^−/−^ mice. (**A**,**B**) RT‐qPCR data of metabolic gene expression in eWAT and iBAT from WT and miR‐34a^−/−^ (KO) mice after 24 weeks on chow vs. high‐fat diet (HFD), normalized to 18s rRNA; *n =* 6 for all groups, except *n =* 5 for KO HFD. All graphs represented as relative quantification (RQ) with RQ_min_ − RQ_max_ values. *P < 0.05, **P < 0.01, ***P < 0.001, ****P < 0.0001, one‐way ANOVA, with Bonferroni's multiple comparisons post‐test.

To investigate the changes in adipose weight and adipocyte size, we examined metabolic gene expression in eWAT *ex vivo*. The following FA and cholesterol metabolism genes' expression was increased in chow‐fed miR‐34a^−/−^ mice compared with WT chow counterparts: *Cd36* (*P =* 0.0036), 3‐hydroxy‐3‐methyl‐glutaryl‐CoA reductase *(Hmgcr*; *P =* 0.0012), liver X receptor (*Lxrα*; *P =* 0.0034), *Pgc‐1*α (*P =* 0.0138), and fatty acid synthase (*Fasn*; *P =* 0.0027). In addition, decreased *Ppar‐γ* (*P =* 0.0471), acetyl‐CoA carboxylase (Acc‐α; *P =* 0.0018), and *Fasn* (*P =* 0.0274) transcripts were observed in miR‐34a^−/−^ mice when fed HFD (Figure 3A). We observed no changes in other eWAT metabolic genes examined (Supporting Information Figure S3A).

Furthermore, we investigated the expression of metabolic genes in iBAT. Interestingly, we observed decreased expression of *Cebpα* (*P <* 0.01) and *Cebpβ* (*P <* 0.01) from both miR‐34a^−/−^ groups and increased expression of *Pgc1*α (*P =* 0.0072) in miR‐34a^−/−^ mice when fed HFD (Figure 3B). However, *Ucp1* expression remained unchanged. Additionally, we saw few changes in hepatic metabolic genes (Supporting Information Figure S3B–D).

### eWAT macrophages from HFD‐fed miR‐34a^−/−^ mice expressed an F4/80^high^ phenotype, with an increase in type 2 cytokines

**Figure 4:**
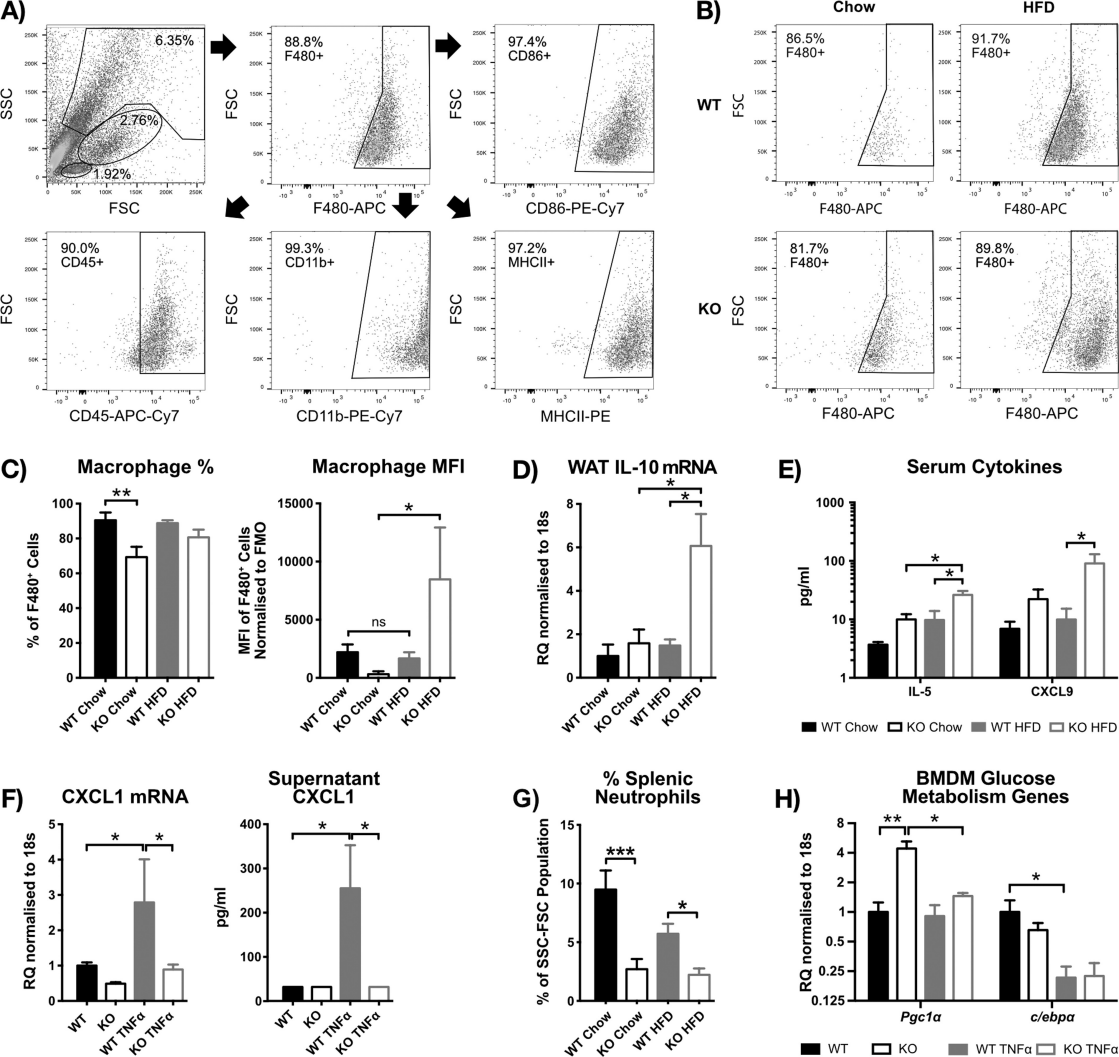
High‐fat diet (HFD) ‐fed miR‐34a^−/−^ mice show an F4/80^high^ phenotype, with increased type 2 cytokines and a reduction in splenic neutrophils. (**A**) Representative dot‐plots showing the gating strategy used to identify macrophages in the SVF from murine epididymal (e)WAT at week 24 in WT and miR‐34a^−/−^ (KO) mice on chow vs. HFD. (**B**) Representative dot‐plots showing the F4/80^high^ macrophage phenotype in KO eWAT after HFD feeding, from the same study as panel A. (**C**) Quantification of panel B, showing median fluorescence intensity (MFI) values representing surface expression and percentage F4/80+ cells in the macrophage FSC‐SSC gate; *n =* 9 for all groups, except *n =* 10 for KO chow. (**D**) RT‐qPCR data of interleukin (IL)‐10 gene expression in eWAT from same study as panel A, normalized to 18s rRNA; *n =* 6 for all groups, except *n =* 5 for KO HFD and WT chow. (**E**) Significant changes from cytokine Luminex data of serum from mice fasted for 16 to 18 h from the same study as panel A; *n =* 6 for KO chow and WT HFD groups and *n =* 5 for WT chow and KO HFD groups. (**F**) RT‐qPCR gene expression and supernatant, cytokine Luminex protein data for CXCL1, from WT and KO *in vitro* bone marrow‐derived macrophages (BMDM) ± 45.45 ng/mL tumor necrosis factor (TNF)‐α for 24 h; *n =* 3. RT‐qPCR data normalized to 18s rRNA. (**G**) FACS quantification of the percentage of neutrophils (CD45^+^ CD11b^+^ F4/80^−^ CD11c^−^Ly6c‐Ly6g^+^) in the FSC‐SSC population from spleens of mice in the same study as panel A. Gating strategy is shown in Supporting Information Figure S4D; *n =* 8 for all groups, except *n =* 4 for WT chow and *n =* 6 for WT HFD. (**H**) RT‐qPCR quantification of peroxisome proliferator‐activated receptor‐y coactivator (PGC)‐1α and *c/ebp*α transcripts in the same samples as panel F; *n =* 3. All graphs represent mean values with SEM, except for RT‐qPCR, represented as relative quantification (RQ) with RQ_min_ − RQ_max_ values. *P < 0.05, **P < 0.01, ***P < 0.001, one‐way ANOVA, with Bonferroni's multiple comparisons post‐test.

Given that miR‐34a transcripts were increased in WT BMDMs stimulated with TNF‐α, they may contribute to the *in vivo* metabolic profile. Therefore, using FACS we gated on the larger, more granular F4/80^+^ cells from WT and 34a^−/−^ eWAT to identify macrophages, after 24 weeks on chow or HFD (Figure 4A). These cells were CD45^+^, CD11b^+^, MHCII^+^, and CD86^+^. Further analysis revealed a population of F4/80^high^ cells within the miR‐34a^−/−^ HFD group not observed in WT (Figure 4B). We observed a significant increase in F4/80 surface expression on miR‐34a^−/−^ cells, when fed HFD (*P =* 0.0497), and a lower F4/80^+^ macrophage content within the eWAT of miR‐34a^−/−^ chow mice, compared with WT chow (*P =* 0.0078) (Figure 4C). There were no changes in the other macrophage M1/M2 surface markers examined (Supporting Information Figure S4A), or M1/M2 genes within the eWAT, except a basal increase in miR‐34a^−/−^ chow eWAT *Nos2* (*P =* 0.0500) and decreased HFD‐fed miR‐34a^−/−^ eWAT *Retnla* (*P =* 0.0006) transcripts, over WT diet controls (Supporting Information Figure S4B). To check whether an increase in cytosolic lipid could contribute to the F4/80^high^ macrophage phenotype in miR‐34a^−/−^ HFD eWAT, we back‐gated on this population for side‐scatter, a measurement of internal complexity, but observed no difference in side‐scatter MFI between any of the groups (Supporting Information Figure S4C).

Bassaganya‐Riera et al. identified a subset of F4/80^high^ ATMs that produced high levels of anti‐inflammatory IL‐10 18. Therefore, we examined eWAT cytokine transcripts and observed increased *IL‐10* in miR‐34a^−/−^ HFD eWAT, compared with WT HFD and miR‐34a^−/−^ chow eWAT (*P <* 0.05) (Figure 4D). Additionally, serum cytokine levels of IL‐5 (*P =* 0.0103) and chemokine CXCL9 (*P =* 0.0303) were increased in HFD‐fed miR‐34a^−/−^ mice, over WT controls (Figure 4E). However, serum IL‐10 was not detected in any group. miR‐34a^−/−^ and WT *in vitro* BMDMs were stimulated with TNF‐α to assess macrophage‐mediated modulation of metabolic and inflammatory pathways. Interestingly, TNF‐α stimulation of miR‐34a^−/−^ macrophages did not upregulate transcripts of the inflammatory, neutrophil chemotactic cytokine *Cxcl1* (*P =* 0.0168), or secreted protein (*P =* 0.0470), as in WT controls (Figure 4F). As a corollary, we observed a reduction in splenic neutrophil percentage (CD45^+^ CD11b^+^ F4/80^−^ CD11c^−^ Ly6c‐Ly6g^+^) in miR‐34a^−/−^ mice from both chow (*P =* 0.0003) and HFD (*P =* 0.0356) groups, compared with WT controls at week 24 of diet (Figure 4G). However, the same was not observed in the eWAT (Supporting Information Figure S4D).

Examination of *Cd36*, *Hmgcr*, *Lxrα*, *Ppar‐γ, Fasn*, and *Acc‐α* transcripts in BMDMs revealed no change (Supporting Information Figure S4E, F). However, higher basal transcript expression of *Pgc1α* (*P =* 0.0024) was observed in miR‐34a^−/−^ BMDMs, which decreased to WT levels upon TNF‐α stimulation (*P =* 0.0139) (Figure 4H). Additionally, other serum and BMDM supernatant cytokines from miR‐34a^−/−^ and WT mice did not change (Supporting Information Figure S5).

### *In vitro* miR‐34a^−/−^ adipocytes showed changes in lipid content

**Figure 5:**
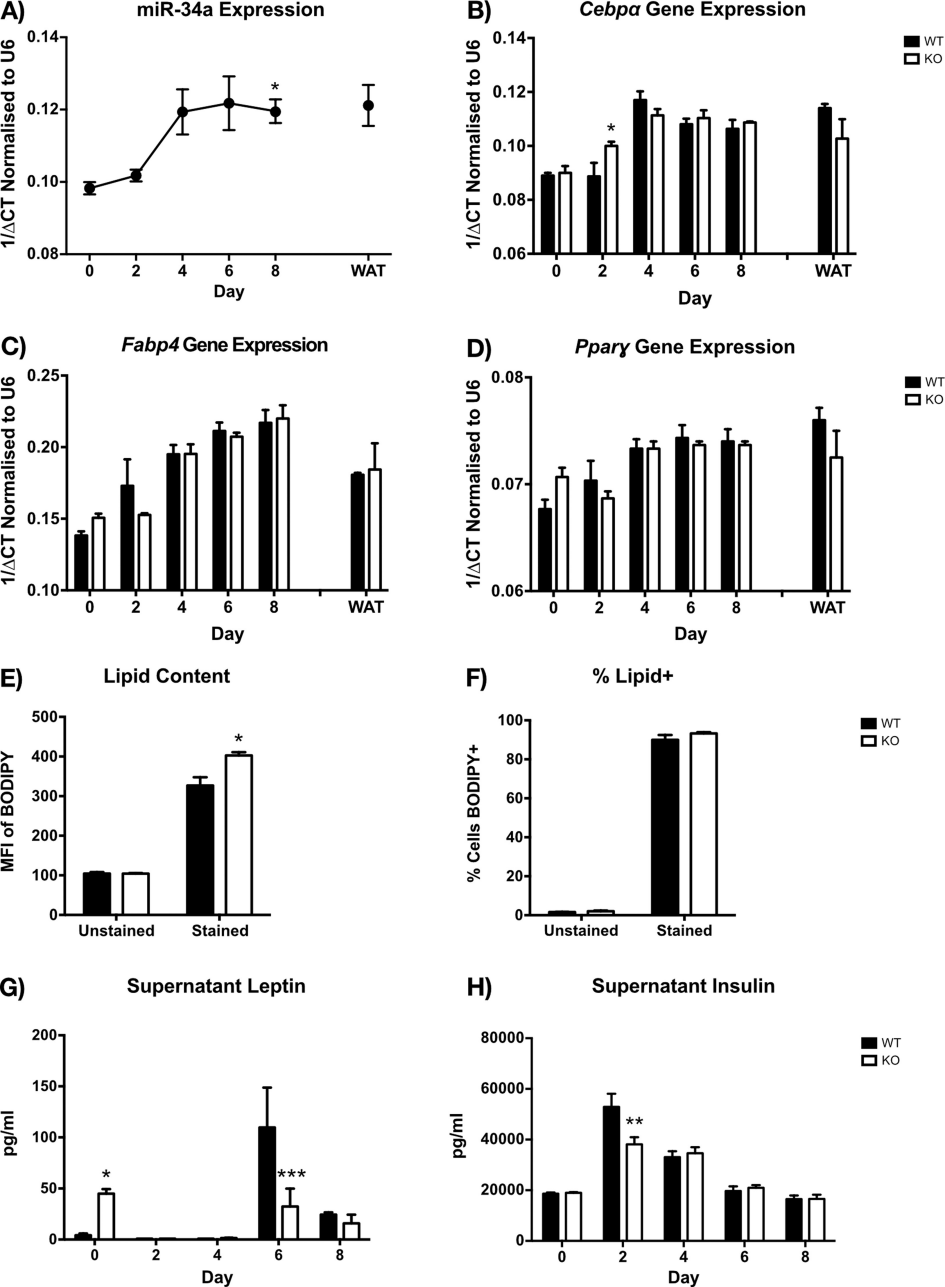
miR‐34a increases over *in vitro* white adipocyte differentiation, and miR‐34a^−/−^ white adipocytes show increased lipid content at day 8. (**A**) RT‐qPCR quantification of miR‐34a transcripts over *in vitro* differentiation of SVF white preadipocytes (day 0–8) from WT murine epididymal (e)WAT, with mature adipocyte fraction (WAT) as a positive control, normalized to RNU6B; three mice were pooled together for each replicate of *n =* 3. (**B**–**D**) RT‐qPCR gene expression over *in vitro* differentiation of SVF white preadipocytes (day 0–8) from miR‐34a^−/−^ (KO) and WT murine eWAT, with mature adipocyte fraction (WAT) as a positive control, normalized to 18s rRNA; three mice were pooled together for each replicate of *n =* 3. (**E**,**F**) Quantification of lipid in day 8 *in vitro* adipocytes by FACS and BODIPY, represented as geometric mean fluorescence intensity (MFI) values representing content, and percentage positive cells; *n =* 3. (**G**,**H**) Adipokine Luminex measurements of leptin and insulin from supernatants of cultures in panels B‐D; *n =* 3. All graphs represent mean values with SEM, except for RT‐qPCR, represented as 1/ΔCt. Statistics were calculated using a two‐way ANOVA, with Bonferroni's multiple comparisons post‐test, or an unpaired Student's *t*‐test for FACS lipid measurements. A one‐way ANOVA and Dunnett's multiple comparisons post‐test was used to calculate statistics for panel A, compared with day 0. *P < 0.05, **P < 0.01, ***P < 0.001.

To examine the function of miR‐34a in adipose tissue, we differentiated primary white and brown adipocytes *in vitro*. We observed significant upregulation of miR‐34a transcripts (*P =* 0.0330) by day 8 of differentiation in WT white adipocyte precursors (Figure 5A), with upregulation of miR‐34b* and 34c* at day 4 (*P <* 0.05), which decreased by day 8 (Supporting Information Figure S6A).

Therefore, we examined differentiation genes in WT and miR‐34a^−/−^ white adipocytes. Examination of *Cebpα*, *Ppar‐γ*, and *Fabp4* over the 8‐day differentiation revealed only a small increase in *Cebpα* expression (*P =* 0.0396) at day 2 in miR‐34a^−/−^ adipocytes (Figure 5B–D). Interestingly, we observed increased lipid content in day 8 miR‐34a^−/−^ white adipocytes (*P =* 0.0280) (Figure 5E) by FACS, with no change in the percentage of lipid‐positive cells (Figure 5F).

Surprisingly, miR‐34a^−/−^ white adipocytes had increased supernatant leptin at day 0 (*P =* 0.0458), with lower levels at day 6 (*P =* 0.0009) of differentiation, compared with WT (Figure 5G). Furthermore, supernatant insulin was decreased at day 2 (*P =* 0.0041) of miR‐34a^−/−^ white adipocyte differentiation, compared with WT (Figure 5H). We observed no change in resistin (Supporting Information Figure S6B). In day 8 brown adipocytes, we did not observe any changes in leptin, insulin, or resistin (Supporting Information Figure S6C). Additionally, obesity proteome array analysis of day 8 supernatants from white and brown adipocyte cultures did not reveal any other cytokine or adipokine differences (Supporting Information Figure S7).

**Figure 6:**
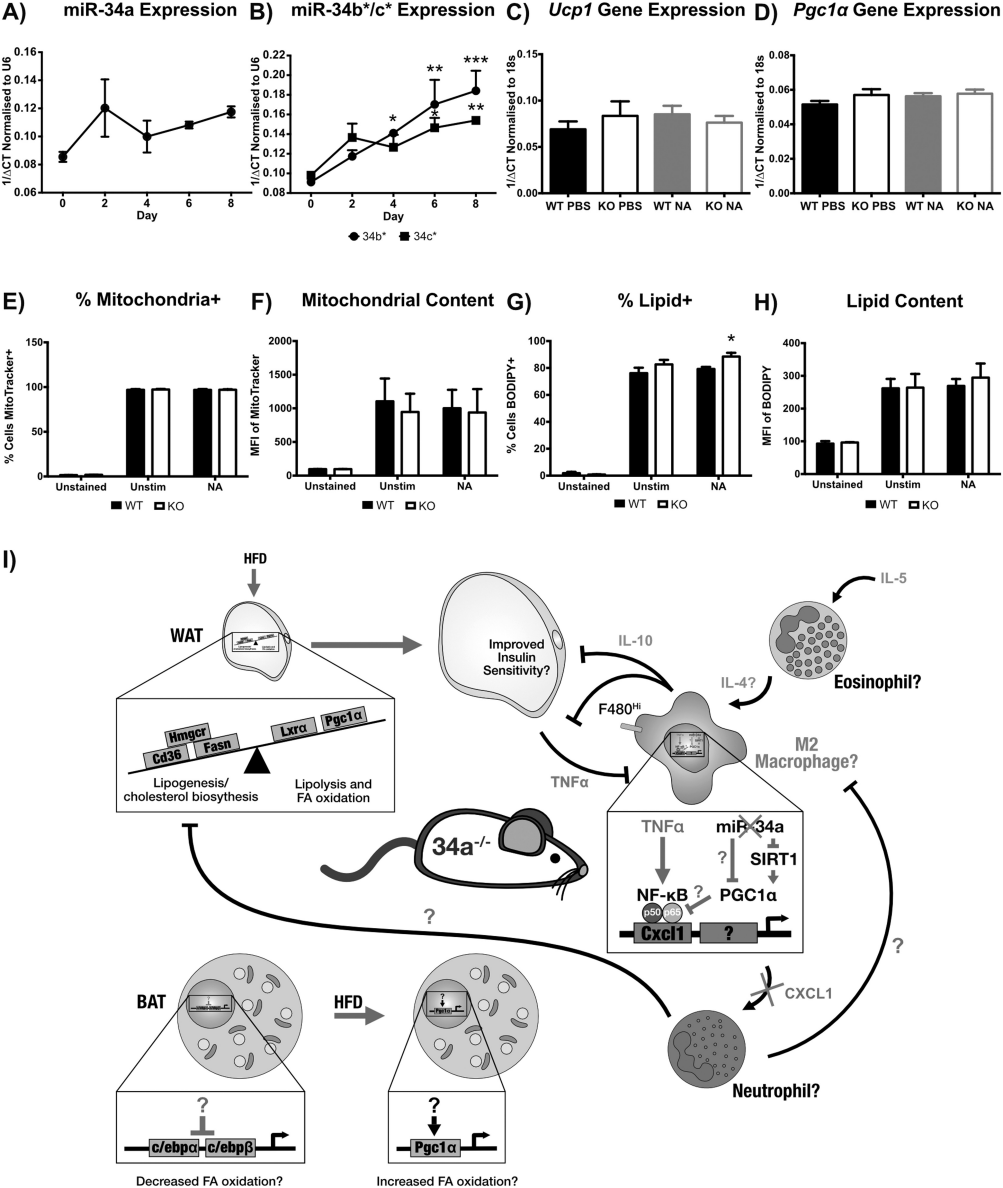
*In vitro* miR‐34a^−/−^ brown adipocytes show an increase in percentage lipid+ cells but no change in mitochondrial markers. (**A**,**B**) RT‐qPCR quantification of miR‐34a, 34b*, and 34c* over *in vitro* WT brown adipocyte differentiation (day 0–8) from intrascapular (i)BAT SVF precursors, normalized to RNU6B; three mice were pooled together for each replicate, *n =* 3–4. (**C**,**D**) RT‐qPCR gene expression at day 8 of *in vitro* differentiation of SVF brown preadipocytes from miR‐34a^−/−^ (KO) and WT murine iBAT, after a 4‐h stimulation with 0.1 µM noradrenaline (NA) or PBS control, normalized to 18s rRNA; three mice were pooled together for each replicate, *n =* 4. (**E**,**F**) Quantification of mitochondria in day 8 *in vitro* brown adipocytes stimulated with 0.1 µM NA or PBS for 4 h, by FACS and MitroTracker Deep Red, shown as geometric mean fluorescence intensity (MFI) values representing content, and percentage positive cells; *n =* 3. (**G**,**H**) Quantification of lipid in day 8 *in vitro* brown adipocytes stimulated with 0.1 µM NA or PBS for 20 h, by FACS and BODIPY, shown as geometric MFI values representing content, and percentage positive cells; *n =* 3. All graphs represent mean values with SEM, except for RT‐qPCR, represented as 1/ΔCt. Statistics were calculated using a one‐way ANOVA, with Bonferroni's multiple comparisons post‐test, or an unpaired Student's *t*‐test for FACS measurements. *P < 0.05, **P < 0.01, ***P < 0.001. (**I**) A diagrammatic representation of the theoretical mechanism in miR‐34a^−/−^ (KO) mice, predisposing them to diet‐induced obesity. The imbalance in WAT metabolic genes caused by chronic overexpression of peroxisome proliferator‐activated receptor‐y coactivator (PGC)‐1α and mitochondrial dysfunction promotes lipid uptake/storage and weight gain when stressed with a high‐fat diet (HFD). As the tissue increases in size there is an increase in inflammatory cytokines, such as tumor necrosis factor (TNF)‐α, which polarize macrophages to an M1 phenotype. However, the KO macrophages are unresponsive to TNF‐α induction of CXCL1 and possibly other pro‐M1 genes, through overexpression of PGC1α inhibiting the NF‐κB subunit p65, suggesting an M2 phenotype. This inhibits the recruitment of neutrophils which could promote an M1 phenotype and inhibit lipogenic processes. The M2 phenotype is further promoted by interleukin (IL)‐5, which can induce eosinophils to produce pro‐M2 IL‐4. IL‐10 likely produced by macrophages can then stimulate adipocytes to be more insulin sensitive and dampen inflammatory processes, reducing pro‐obesity processes. Inhibition of key thermogenic genes in BAT predisposes the mice to obesity development, with overcompensation by the induction of PGC1α during HFD feeding.

To further explore the metabolic changes in miR‐34a^−/−^ iBAT, we examined miR‐34a expression over *in vitro* differentiation of WT brown adipocyte precursors, but found no change in miR‐34a (Figure 6A). However, we observed increases in miR‐34b* at day 4 (*P =* 0.0305) and both miR‐34b* and 34c* at day 6 (*P <* 0.05) and day 8 (*P <* 0.01) of differentiation (Figure 6B).

WT and miR‐34a^−/−^ day 8 brown adipocytes were stimulated with noradrenaline to examine the effect of β‐adrenergic stimulation on *Ucp1* and *Pgc1α* transcripts. However, we observed no difference in *Ucp1* and *Pgc1α* transcript expression (Figure 6C, D). Additionally, we quantified cellular mitochondrial content by FACS but found no difference (Figure 6E, F). However, FACS analysis identified an increase in the percentage of noradrenaline‐stimulated, miR‐34a^−/−^ lipid‐positive brown adipocytes (*P =* 0.0445) (Figure 6G), with no difference in cellular lipid content (Figure 6H).

We examined the expression of key brown adipocyte differentiation genes, but found no difference in *Pgc1α*, *Ucp1*, *Cebpα*, *Cebpβ*, or *Ppar‐γ* transcripts between WT and miR‐34a^−/−^ brown adipocytes (Supporting Information Figure S8A). Finally, we observed no change in white adipocyte *Cd36*, *Hmgcr*, *Lxrα*, *Pgc1α, Fasn*, and *Acc‐α* metabolic genes (Supporting Information Figure S8B).

## Discussion

The regulation of chronic, low‐grade inflammation of adipose tissue during obesity is poorly understood and this study aimed to address the role of miR‐34a in this process. Our findings showed that miR‐34a^−/−^ mice were heavier than WT controls and gained more weight when challenged with a HFD, suggesting they were more susceptible to weight gain. miR‐34a^−/−^ eWAT showed smaller, more numerous adipocytes, with a lower tissue mass on chow that increased to WT levels on HFD. It is surprising that there was no difference in these characteristics between HFD‐fed miR‐34a^−/−^ and WT mice, despite a clear difference in body weight. With no change in liver weight, this could suggest increases in other WAT depots (e.g., scWAT), BAT, or muscle tissue. One gene that was increased in all the miR‐34a^−/−^ tissues examined was *Pgc1α*, suggesting that it could have broad regulation by miR‐34a. During preparation of this article, Fu et al. showed that miR‐34a could increase acetylation of PGC1α, decreasing its activation, by inhibiting SIRT1 activity and FGF21 signaling in HFD‐fed murine eWAT and 3T3L1 adipocytes, promoting adipose browning 19. Correlating with our results, the authors observed increased transcripts of *Pgc1*α in eWAT and BAT after lentiviral‐mediated inhibition of miR‐34a in WAT and BAT of male, HFD‐fed BALB/c and C57BL/6 mice. However, they also observed a reduction in body weight, and visceral (epididymal and peritoneal) and scWAT, with improved glucose tolerance. Earlier studies have also shown anti‐miR‐34a administration to HFD‐fed mice improved systemic glucose and insulin tolerance, and liver phenotype 20, 21. All these studies used acute miR‐34a inhibition in established murine obesity, which is milder than the 24‐week HFD feeding used here. These differences with our model are likely due to compensatory effects of using a full‐body KO. It seems paradoxical that miR‐34a ablation causes an upregulation in *Pgc1α* transcripts, but at the same time promotes weight gain. However, full‐body Pgc1α KO mice were shown to lose weight, rather than gain weight as expected, and skeletal muscle‐specific murine Pgc1α overexpression (MPGC‐1α TG) caused muscle adipose accumulation, atrophy, and decreased ATP synthesis and content, indicative of mitochondrial respiratory uncoupling 22, 23.

Respiratory uncoupling by UCPs has been linked to increased FA oxidation 24. It may seem counterproductive that in miR‐34a^−/−^ eWAT we saw basal increases in genes involved in lipid uptake: *Cd36, de novo* lipogenesis: *Fasn*, and cholesterol biosynthesis: *Hmgcr*, which caused increases in adipocyte total and free cholesterol, associated with omental adipocyte hypertrophy in primates 25, 26, 27. However, MPGC‐1α TGs have increased expression of *Cd36* and *Fasn*, with *Fasn* expression driven by PGC1α's increase in *Lxrα* expression, and increased intracellular lipid 28. The authors suggest that this response “refuels” the cell's lipid stores for continued FA oxidation. Therefore, there may be an imbalance in lipolysis/FA oxidation vs. lipid uptake/lipogenesis, pushing metabolism to the latter in adipocytes of miR‐34a^−/−^ mice, due to chronic PGC1α upregulation, which predisposes these mice to weight gain when stressed with a HFD. Additionally, this would explain why miR‐34a^−/−^ mice are heavier before HFD feeding. In fact, when MPGC‐1α TGs were fed HFD they developed insulin resistance and were not protected from diet‐induced obesity, showing increased lipid uptake and lipogenesis genes in skeletal muscle, including *Cd36*, and no change in resting serum insulin, triglycerides, cholesterol, glucose, and FFAs 29. The increased lipid content observed in mature *in vitro* miR‐34a^−/−^ white adipocytes supports this metabolic imbalance. Additionally, the increase in miR‐34a in WT adipocytes over the time course of differentiation suggests that it may regulate these processes in mature adipocytes, due to the lack of change in differentiation makers in miR‐34a^−/−^ white adipocytes. The decreases in *Ppar‐γ*, *Fasn,* and *Acc‐α* eWAT transcripts upon HFD feeding in miR‐34a^−/−^ mice, could be evidence of the balance being shifted back to FA oxidation to regain lipid homeostasis, by other regulatory systems. This could be assisted by the upregulation of *Pgc‐1α* mRNA in HFD‐fed miR‐34a^−/−^ BAT, which could compensate for the reduced *c/ebpα* and *β* mRNA. Both c/EBPα and β transactivate UCP1, and c/EBPβ is essential for brown adipocyte differentiation and thermoregulation 30, 31. Unfortunately, we did not see these differences *in vitro* with β‐adrenergic stimulation, but other stimuli could be involved *in vivo*. Furthermore, PGC1α has been shown to be a predicted target of miR‐34a in humans, but not mice, using miRWalk 32. Therefore, the *in vivo* phenotype of miR‐34a^−/−^ mice could be attributed to PGC1α being a direct target of miR‐34a or indirectly through SIRT1/FGF21.

Our study showed that miR‐34a^−/−^ ATMs have an altered phenotype, and deficiency in inflammatory TNF‐α responses *in vitro*. Flow cytometric analysis identified F4/80^high^ CD45^+^ MHCII^+^ CD11b^+^ CD86^+^ macrophages within eWAT of HFD‐fed miR‐34a^−/−^ mice and a reduced F4/80^+^ macrophage population in chow‐fed miR‐34a^−/−^ mice. F4/80 is a glycoprotein specific for murine macrophages, with expression increasing as macrophages mature, but its specific function remains unknown. However, Lin et al. showed that F4/80 has a role in generating CD8^+^ regulatory T cells 33. F4/80^high^ ATMs have previously been observed in murine WAT, but have been shown to be in equal proportions F4/80^low^:F4/80^high^ in obese *db/db* WAT and greater proportions of F4/80^high^ ATMs in diet‐induced obese WAT 18, 34. Bassaganya‐Riera et al. showed that these F4/80^high^ ATMs expressed higher levels of MHCII, CX3CR1, CD11c, CCR2, and PPAR‐γ in *db/db* mice, with increased intracellular IL‐6, TNF‐α, CCL2, and IL‐10, which increased further upon LPS‐stimulation 18. These reports support our observations of increased *IL‐10* mRNA in HFD‐fed miR‐34a^−/−^ eWAT, correlating with the ATM F4/80^high^ phenotype. The increase in IL‐10 suggests an M2‐like phenotype. Corollary, the *in vitro* data showing ablated upregulation of CXCL1 in TNF‐α‐stimulated miR‐34a^−/−^ BMDMs, suggests a deficiency in TNF‐α signaling. Additionally, the upregulation of miR‐34a transcripts in TNF‐α‐stimulated BMDMs, supports the possibility of miR‐34a regulating the TNF‐α pathway. This has been previously suggested by Jiang et al. with contradictory results using LPS‐stimulated RAW264.7 macrophages, showing miR‐34a inhibits TNF‐α expression by targeting the NF‐κB p50‐subunit activator NOTCH1 12. However, we showed that *Pgc1α* was upregulated in unstimulated miR‐34a^−/−^ *in vitro* macrophages, which others have shown represses TNF‐α‐induced proinflammatory cytokine production by reducing RelA/p65 phosphorylation, inhibiting NF‐κB signalling in murine skeletal muscle 35. Paradoxically, Bassaganya‐Riera et al. suggested that F4/80^high^ macrophages are M1‐like, despite expressing high levels of IL‐10 and PPAR‐γ. Furthermore, we observed a reduction in the M2 marker *Retnla* in HFD‐fed miR‐34a^−/−^ eWAT and an increase in the M1 marker *Nos2* in chow‐fed miR‐34a^−/−^ eWAT. Therefore, it is unclear whether miR‐34a^−/−^ macrophages have a pro‐ or anti‐inflammatory phenotype, but their phenotype could be altered by dysregulation of PGC1α.

Systemically, we observed characteristics of a type 2 immune response in miR‐34a^−/−^ mice with increased serum IL‐5 protein during HFD feeding and a reduction in splenic neutrophils observed in both miR‐34a^−/−^ groups. Interestingly, neutrophil‐specific elastase KO mice have increased M2‐like ATMs and increased transcripts of *Acc‐α* and *Fasn* within WAT 17. However, we did not see the same decrease in eWAT neutrophils. Importantly, a type 2 immune response has been reported to promote increased insulin sensitivity, glucose uptake, and anabolism in white adipocytes, but an overall protective effect from obesity and T2D 16, 36, 37. Therefore, a type 2 immune response in HFD‐fed miR‐34a^−/−^ mice may be initiated as a compensatory effect during overnutrition, to regain metabolic homeostasis. A study limitation is that we did not examine miR‐34a expression in BMDMs from HFD‐fed mice or TNF‐α‐stimulated adipocytes.

In summary, this study has demonstrated that miR‐34a regulates the development of diet‐induced obesity in mice, with miR‐34a^−/−^ mice showing a susceptibility to weight gain, likely through dysregulation of PGC1α (Figure 6I). It is clear that the role of miR‐34a in metabolism is complex and likely to vary depending on context of treatment and model, with potentially contrasting roles in different cell types. miRNAs' fine‐tuning of gene expression results in subtle changes in protein synthesis, making it complex to follow up *in vivo* observations with complete mechanistic data *in vitro*, as seen here. However, this study has highlighted that chronic miR‐34a inhibition, as a treatment for obesity, may have undesirable metabolic and immunoregulatory consequences. Further research is required to fully elucidate the metabolic and inflammatory pathways regulated by miR‐34a.

## Supporting information

Supporting Information
