# Supplementary material for: miR‐34a−/− mice are susceptible to diet‐induced obesity
Source: Obesity (Silver Spring). 2016 Jul 5;24(8):1741–51. doi: 10.1002/oby.21561 (PMC4979678; doi:10.1002/oby.21561)
Supplement: Supplementary file 1 — Supporting Information [file OBY-24-1741-s001.docx]

# Supplementary Information: miR-34a^-/-^ mice are susceptible to diet-induced obesity

**Christopher. A. Lavery^1^, Mariola Kurowska-Stolarska^2^, William M. Holmes^3^, Iona Donnelly^1^, Muriel Caslake^1^, Andrew Collier^4^, Andrew. H. Baker^1^, Ashley. M. Miller^1*^**

1. Institute of Cardiovascular & Medical Sciences, College of Medical, Veterinary and Life Sciences, University of Glasgow, Glasgow, United Kingdom.

2. Institute of Infection, Immunity and Inflammation, College of Medical, Veterinary and Life Sciences, University of Glasgow, Glasgow, United Kingdom.

3. Glasgow Experimental MRI Centre, Institute of Neuroscience and Psychology, University of Glasgow, Glasgow, United Kingdom.

4. Ayr Hospital, National Health Service: Ayrshire & Arran, Ayr, United Kingdom.

**Keywords:** Obesity, adipocyte, macrophage, inflammation, miR-34a.

**Running Title:** miR-34a^-/-^ mice are susceptible to obesity

^*^**Corresponding author:** Ashley M. Miller ([Ashley.Miller@glasgow.ac.uk](mailto:Ashley.Miller@glasgow.ac.uk))

Institute of Cardiovascular & Medical Sciences, College of Medical, Veterinary and Life Sciences, University of Glasgow, Glasgow, G12 8TA, UK. Tel. 0141 330 2418.

# Supplementary Methods

## Murine Metabolic Cage Studies

For metabolic cage (Techniplast, Milan, Italy: 3700M022) studies, mice received a 3-hour acclimatization a week before. Food consumption and excretion measurements were taken from WT or miR-34a^-/-^ mice fed chow or HFD for 9 weeks, over 24-hours.

## Murine Tissue Collection

Mice were terminally anaesthetised using an I.P. injection of Avertin (3 g 2,2,2-Tribromoethanol and 3 ml 2-methyl-2-butanol stock, 1:40 in PBS). Metabolic cage mice were euthanized by terminal CO_2_. Whole blood was collected by cardiac puncture for serum, and tissues collected for further analysis.

## *in vitro* Adipocyte Cultures

epididymal (e)WAT or intrascapular (i)BAT from 5-7-week-old, male mice was collected in wash DMEM (1% penicillin/streptomycin). This was dissociated and incubated at 37°C for 30 minutes in 0.2% Collagenase Ⅱ (Sigma-Aldrich, Gillingham, UK) and collagenase-hepes buffer: 100 mM HEPES, 120 mM NaCl, 4.80 mM KCl, 1 mM CaCl_2_•2H_2_O, 4.5 mM glucose, and 1.5% albumin in dH_2_O; pH 7.4. Digest was passed through a 70 μm filter (Corning, Amsterdam, The Netherlands) and incubated at 4°C for 15 minutes to isolate the stromal vascular fraction (SVF). SVF cells were cultured at 1x10^6^ cells/well in chamber slides with complete DMEM: 10% FCS, 4 mM L-Glutamine, 1% penicillin-streptomycin, 10 μg/ml Insulin (Sigma-Aldrich: I9278), 25 μg/ml Na Ascorbate, 10 mM Hepes, and 1 μM Rosiglitazone (Enzo Life Sciences, Exeter, UK: ALX-350-125-M025), at 37°C with 5% CO_2_ for 8 days. Brown adipocyte day 8 *Ucp1* and *Pgc1α* gene expression, was quantified following a stimulation with 0.1 µM noradrenaline (Sigma-Aldrich) for 4-hours, or 20 hours for mitochondrial and lipid content analysis.

## *in vitro* BMDM Cultures

Bone marrow was isolated from the femur/tibia of female, WT and miR-34a^-/-^ mice, with RPMI wash media (1% Penicillin-Streptomycin) passed through a 70 µm filter. Cells were resuspended in 2 ml RBC Lysis Buffer (Sigma-Aldrich) for 2 minutes, then washed and cultured at 2x10^6^ cells/90 mm petri dish, in complete RPMI (10% FCS, 1% Penicillin-Streptomycin, 2 nM L-glutamine) with 50 ng/ml M-CSF (PeproTech, London, UK: 315-02). Cells were differentiated over 6 days at 37°C with 5% CO_2_. For stimulations, macrophages were plated at 0.5x10^6^ cells/well, with ±45.45 ng/ml TNFα (PeproTech: 315-01A) for 24 hours.

## Serum Lipid Analysis

Serum triglyceride (TG; Randox, Crumlin, UK: TR210), glycerol (Randox: GY105), total cholesterol (Randox: CH200) and High-density lipoprotein (HDL; Roche: 03045935) were measured using an ILAB 600 clinical chemistry analyzer. True TGs were calculated by: True TGs = TGs mmol/L – glycerol mmol/L.

Serum and *in vitro* supernatant adipokines and cytokines were measured by a Mouse Adipokine Milliplex (Millipore-Merck, Livingston, UK: MADKMAG-71K), and Mouse Cytokine 20-Plex (Life Technologies, Paisley, UK: LMC0006), respectively. Plates were analyzed with Bio-Rad’s Bio-Plex 100 Luminex analyzer. Values below the cutoff were replaced with half the lowest standard or lowest extrapolated value, for statistics.

## Histology

Tissues were fixed in 4% paraformaldehyde, processed, and embedded in paraffin. Anonymized eWAT sections were stained with haematoxylin and eosin, and images taken at 40X. Images were analyzed using Fiji 2 software (SciJava consortium). Cell area and number was quantified using Analyze Particles on Threshold adjusted images with “Precentile” preset, after H&E Colour Deconvolution. Images were batch processed and average taken from ≥5 random fields/section.

**MRS**

Mouse body fat percentage was measured by whole body Magnetic Resonance Spectroscopy (MRS), as previously described (1), giving values for percentage body fat. Measurements were made using a 7T Bruker Biospec system (Karlsruhe, Germany).

**Histology**

*In situ* hybridization (ISH) was carried out as previously described (2). 12.5 nM Exiqon (Vedbaek, Denmark) probes hsa-miR-34a (38487-15; ACAACCAGCTAAGACACTGCCA) and Scramble-miR control (99004-15; GTGTAACACGTCTATACGCCCA) were hybridized at 55°C. The AP substrate was developed for 1 hour, and images taken at 10 and 40X.

**Gene Expression**

RNA was extracted with 1 ml Qiazol (Qiagen, Manchester, UK) and RNeasy Mini Kit (Qiagen). cDNA was generated using miScript Reverse Transcription (RT) 2 Kit (Qiagen) for gene expression, and TaqMan® MicroRNA RT Kit (Life Technologies) for miRNA expression. miRNA reverse transcription reactions were multiplexed using Life Technologies protocol: 4465407.

Gene expression was quantified with 1-10 ng cDNA, using Life Technologies’ TaqMan® Gene Expression Master mix and TaqMan® Gene Expression Assays: *Table M1*. miRNA expression was quantified using Life Technologies’ TaqMan® Universal Master mix 2, no UNG with TaqMan® microRNA Assays: *Table M1*. Data was acquired using Sequence Detection Software (SDS) 2.3 software and a 7900HT Fast Real-Time PCR System (Life Technologies).

**Flow Cytometry**

eWAT SVF cells or splenocytes (0.5x10^6^ cells/panel) were washed in 300μl FACS buffer (DPBS, 2% FCS, and 2mM EDTA) and incubated with 50 μl Fc block (BD, Oxford, UK: 553142) at 4°C for 15 minutes. Cells were stained with the anti-mouse antibodies in *Table M2* for 30 minutes. Cell marker expression was quantified using a BD Canto 2. Data was analyzed using FlowJo v.10.0.7 software, with gates set on Fluorescence Minus One (FMO) controls. Median fluorescence intensity (MFI) values were normalized to FMOs.

For lipid and mitochondrial content analysis, cells in FACS buffer were incubated with 0.1 µg/ml BODIPY 493/503 (Life Technologies) or 50 nM MitoTracker Deep Red FM (Life Technologies) for 30 minutes at 37°C.

**Statistics**

All graphs were produced using Prism (GraphPad Software, Inc. v4), with data graphed as mean values of ≥3 biological replicates, averaged from ≥2 technical repeats. Error bars are calculated as SEM or RQmax and RQmin. Statistics were calculated by Student’s t test, or One-way or Two-way ANOVA.

| Probe | Cat no. | Probe | Cat no. | Probe | Cat no. |
| --- | --- | --- | --- | --- | --- |
| 18s rRNA | Mm03928990_g1 | Cyp7a1 | Mm00484150_m1 | Fasn | Mm00662319_m1 |
| Fabp4 | Mm00445878_m1 | Arg1 | Mm00475988_m1 | Cd36 | Mm01135198_m1 |
| Pparɣ | Mm01184322_m1 | Pck1 | Mm01247058_m1 | Ccl2 | Mm00441242_m1 |
| Cebpα | Mm01265914_S1 | Chi3l3 | Mm00657889_mH | Lpl | Mm00434764_m1 |
| Sirt1 | Mm00490758_m1 | Il-6 | Mm00446190_m1 | Il-10 | Mm01288386_m1 |
| Accα | Mm01304257_m1 | Nos2 | Mm01309897_m1 | miR-34a | 000426 |
| Cpt1a | Mm01231183_m1 | Retnla | Mm00445109_m1 | miR-34a* | 465771_mat |
| Crot | Mm00470079_m1 | TNFα | Mm00443260_g1 | miR-34b | 002617 |
| Hmgcr | Mm01282499_m1 | Pparδ | Mm01305434_m1 | miR-34b* | 002618 |
| Lxrα | Mm00443451 | Cebpβ | Mm00843434_s1 | miR-34c | 000428 |
| Srebf1 | Mm00550338_m1 | Pgc1α | Mm00447183_m1 | miR-34c* | 002584 |
| Cxcl1 | Mm04207460_m1 | Ucp1 | Mm01244861_m1 | RNU6B | 001973 |

**Table M1: qRT-PCR TaqMan® Assay Information**

| **Antibody** | **Supplier** | **Cat no.** | **Antibody** | **Supplier** | **Cat no.** |
| --- | --- | --- | --- | --- | --- |
| F480-APC | eBio | 17-4801-82 | CD4-PE | BD | 553048 |
| CD45-APC-Cy7 | BD | 561037 | TLR2-PE | eBio | 12-9021-82 |
| CD86-PE-Cy7 | BD | 560582 | CD8a-APC | eBio | 17-0081-82 |
| CD11b-PE-Cy7 | BD | 561098 | MHC Class Ⅱ | BD | 558593 |
| CD80-PerCP-Cy5.5 | BD | 560526 | Ly-6g/c-PerCP-Cy5.5 | BD | 561103 |
| CD69-PerCP-Cy5.5 | BD | 561931 | CD3-FITC | BD | 555274 |
| CD19-PerCP-Cy5.5 | BD | 551001 | CD11c-FITC | BD | 557400 |
| CD206-PE | Omni |  |  |  |  |

**Table M2: FACS Antibody Information**

# Supplementary Results Figures


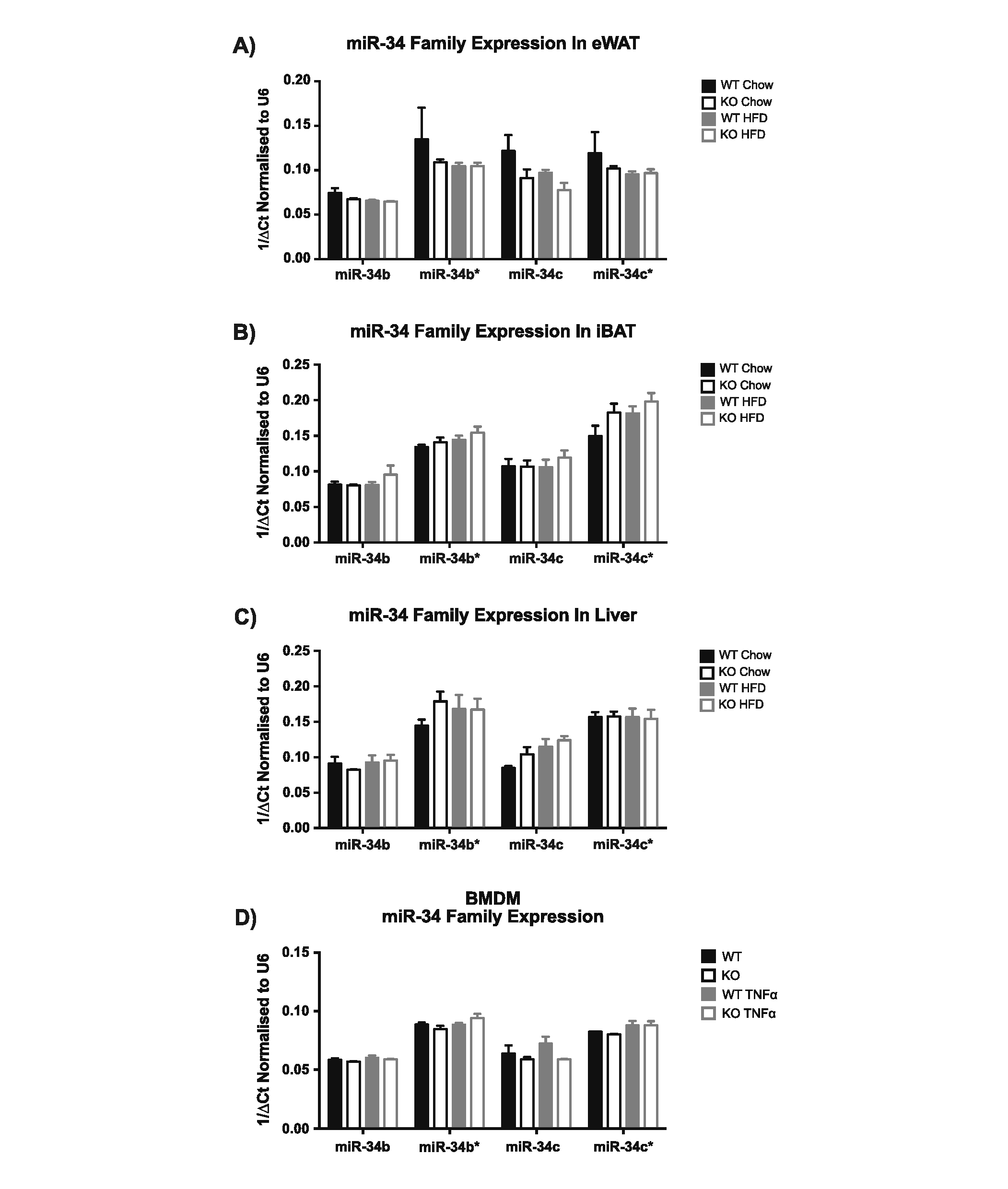


### Figure S1

RT-qPCR data showing the transcript expression of the other miR-34 family members (34b, 34b*, 34c, and 34c*) in the WAT (A; n=3-4), BAT (B; n=6), and Liver (C; n=5-6) of WT and miR-34a-/- (KO) mice at week-24 of chow Vs. HFD. (D) shows transcript expression of the miR-34 family in WT and KO *in vitro* bone-marrow derived macrophages (BMDM) +/- 45.45 ng/ml TNFα for 24 hours, normalized to RNU6B; n=3. For all, undetermined values were replaced with a Ct = 40 for graphing, with 34b and 34c showing very low to no expression. Data is represented as 1/∆Ct, normalized to RNU6B expression, with SEM. Statistics were calculated by One-way ANOVA, with Bonferronni’s multiple comparisons post-test.


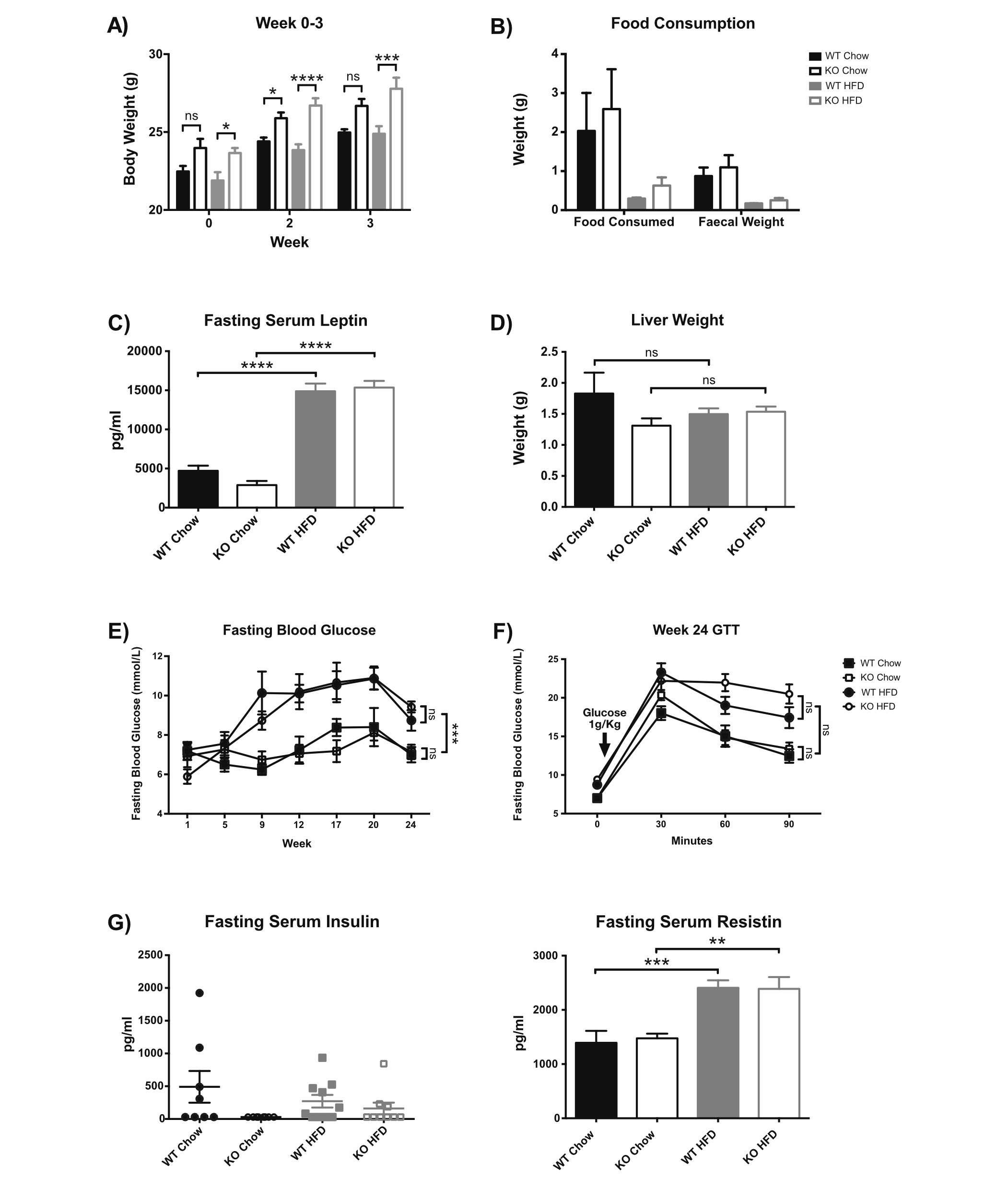


### Figure S2

(A) Comparison of WT and miR-34a^-/-^ (KO) mouse body weight at weeks 0-3 on chow Vs. HFD for 24 weeks; n=8-10. Data (C-G) is from the same mice used here. (B) Metabolic cage data showing food consumption and excretion in 24 hours from WT and miR-34a-/- (KO) mice, after 9 weeks on chow Vs. HFD; n=3 for all groups. (C) Adipokine luminex measurements of serum leptin following a 16-18 hour fast; n=8 for WT and KO Chow groups, n=9 for WT HFD and n=10 for KO HFD groups. (D) Weight of excised liver at week-24 of *in vivo* study; n=10 for miR-34a^-/-^ (KO) chow and WT HFD, n=9 for WT chow and KO HFD. (E) Monthly, fasting blood glucose measurements after a 16-18hour fast; n=10 for KO Chow and WT HFD, and n=9 for WT chow and KO HFD groups. (F) GTT fasting blood glucose measurements following a 16-18 hour fast and I.P. injection of 1g/kg body weight glucose (arrow) after 0 minutes; n=9 for all groups, except n=10 for WT HFD group. (G) Adipokine luminex measurements of insulin and resistin from fasting serum; n=8 for WT and KO Chow groups, n=9 for WT HFD and n=10 for KO HFD groups. All graphs represent mean values with SEM. *P<0.05, **P<0.01, ***P<0.001, ****P<0.0001 One-way ANOVA, with Bonferronni’s multiple comparisons post-test.

| **Group** | **Cholesterol (mmol/L)** | **HDL (mmol/L)** | **Triglycerides (mmol/L)** | **Free-Glycerol (mmol/L)** | **True TG (mmol/L)** |
| --- | --- | --- | --- | --- | --- |
| **WT Chow** | 1.433 (±0.14) | 1.461 (±0.13) | 0.266 (±0.2) | 0.255 (±0.02) | 0.053 (±0.01) |
| **KO Chow** | 1.301 (±0.33) | 1.292 (±0.29) | 0.244 (±0.03) | 0.241 (±0.03) | 0.024 (±0.01) |
| t test (P=) | 0.771 | 0.670 | 0.556 | 0.735 | 0.287 |
| **WT HFD** | 3.334 (±0.25) | 2.887 (±0.19) | 0.229 (±0.03) | 0.208 (±0.02) | 0.050 (±0.01) |
| **KO HFD** | 3.288 (±0.17) | 2.876 (±0.11) | 0.192 (±0.03) | 0.204 (±0.02) | 0.027 (±0.01) |
| t test (P=) | 0.887 | 0.976 | 0.754 | 0.885 | 0.305 |

### Table S1

Representing serum lipid and cholesterol measurements from WT and miR-34a^-/-^ mice on chow Vs. HFD for 24 weeks, following a 16-18 hour fast; High density lipoprotein (HDL), Triglycerides (TG); n=5-10. Negative values on calculating true TG vales were excluded. Statistics were calculated between WT and KO groups by unpaired student’s t-test.


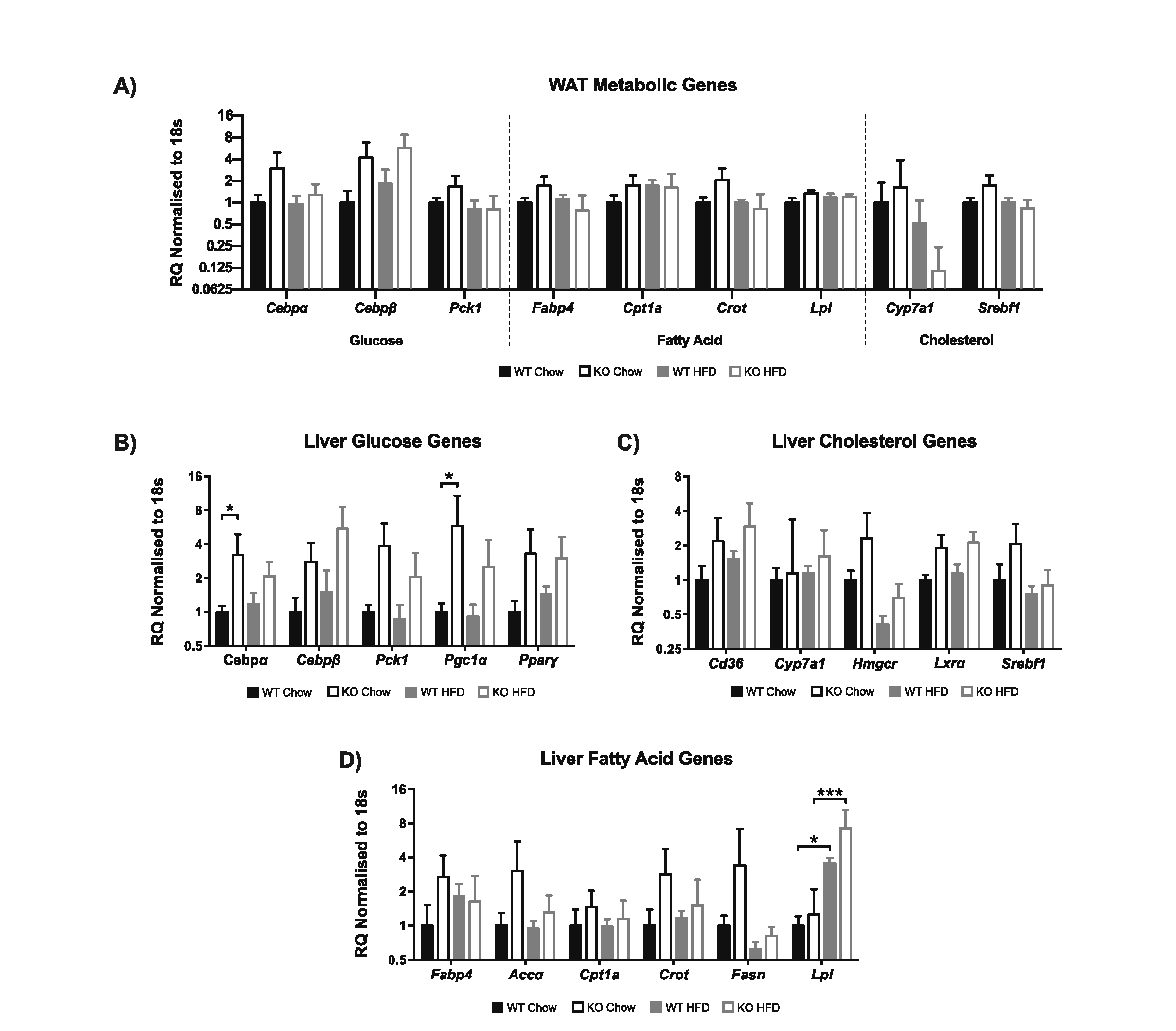


### Figure S3

Metabolic RT-qPCR gene expression data from epididymal white adipose tissue (eWAT; (A)) and liver (B-D), of WT and miR-34a^-/-^ (KO) mice after 24 weeks on chow Vs. HFD. For all groups n=6, except KO HFD n=5. Data is mean values represented as relative quantification (RQ) with RQ_max_ and RQ_min_ values. *P<0.05, ***P<0.001 One-way ANOVA, with Bonferronni’s multiple comparisons post-test.


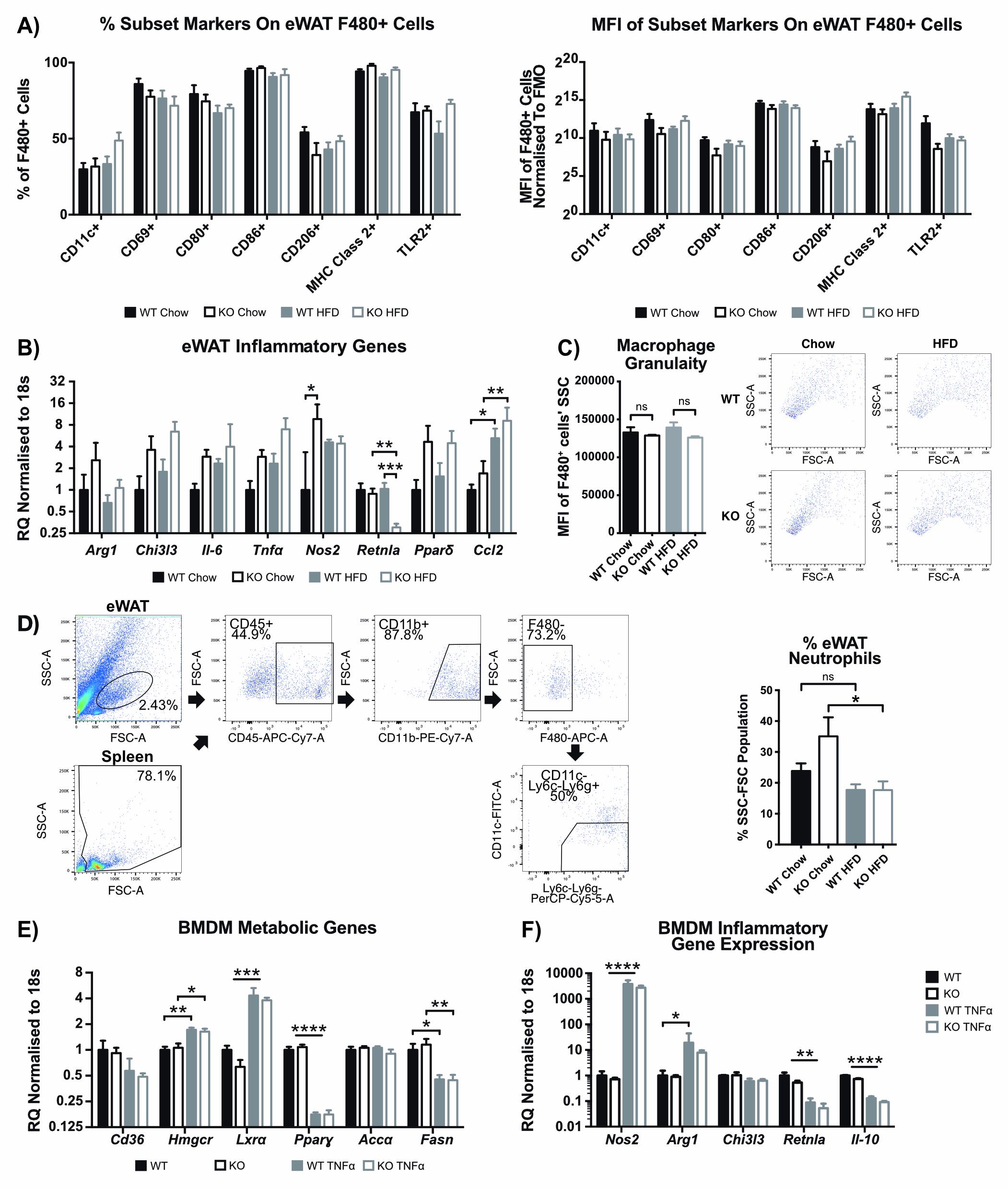


### Figure S4

(A) Macrophage subset marker expression on F480^+^ cells (macrophages) from *ex vivo* eWAT from WT and miR-34a^-/-^ (KO) mice on week-24 of chow Vs. HFD, represented as percentage of positive cells and median fluorescence intensity (MFI) for surface expression. MFI is normalized to the florescence minus one (FMO) control; n=9 for all, except n=10 for KO Chow. (B) Inflammatory RT-qPCR gene expression data from epididymal white adipose tissue (eWAT) of WT and KO mice after 24 weeks on chow Vs. HFD; for all groups n=6, except KO HFD n=5. Data is represented as relative quantification (RQ) with RQ_max_ and RQ_min_ values. (C) Quantification of F480^+^ macrophage granularity, based on side scatter (SSC) MFI from back-gating on the F480^+^ population from the same samples as (A), and representative SSC-FSC dot plots; n=9-10. (D) Showing FACS gating strategy for eWAT and splenic neutrophils, with quantification of the percentage of CD45^+^ CD11b^+^ F480^-^ CD11c^-^ Ly6c-Ly6g^+^ (neutrophil) cells, in the SSC-FSC population, within the eWAT as in (A); n=4 WT chow, n=6 WT HFD, and n=8 for KO groups. (E-F) RT-qPCR quantification of metabolic and inflammatory genes in WT and KO *in vitro* bone-marrow derived macrophages (BMDM) +/- 45.45 ng/ml TNFα for 24 hours; n=3. Data is represented as RQ with RQ_max_ and RQ_min_ values. *P<0.05, **P<0.01, ***P<0.001, ****P<0.0001 One-way ANOVA, with Bonferronni’s multiple comparisons post-test.


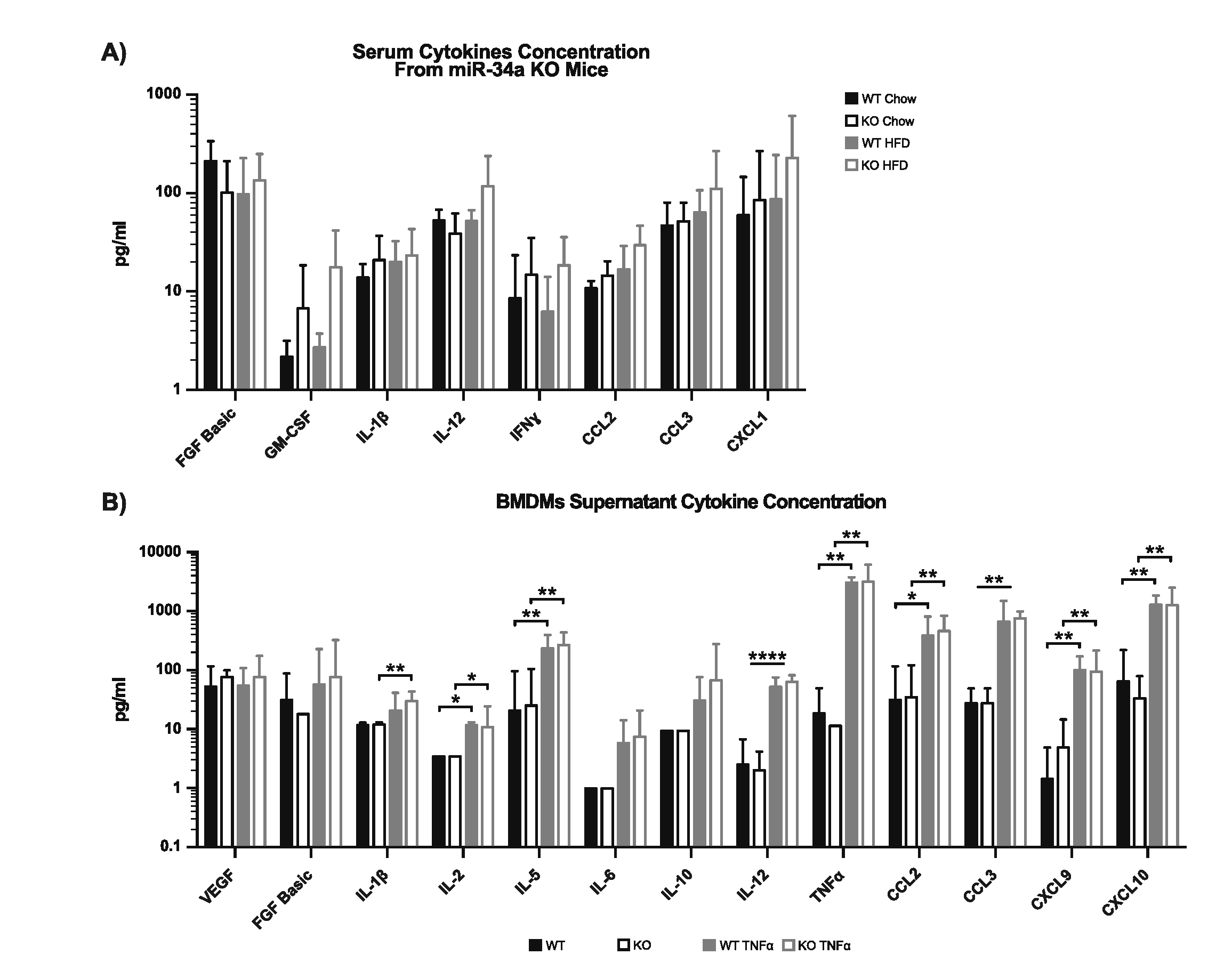


### Figure S5

(A) Showing Luminex cytokine data from the serum of WT and miR-34a^-/-^ (KO) mice after 24 weeks on chow Vs HFD; n=5 for WT chow and KO HFD groups, and n=6 for others. (B) Luminex cytokine data from supernatants of WT and KO *in vitro* bone-marrow derived macrophages (BMDM) +/- 45.45 ng/ml TNFα for 24 hours; n=3. Data is represented as mean values with SEM. *P<0.05, **P<0.01, ****P<0.0001 One-way ANOVA, with Bonferronni’s multiple comparisons post-test.


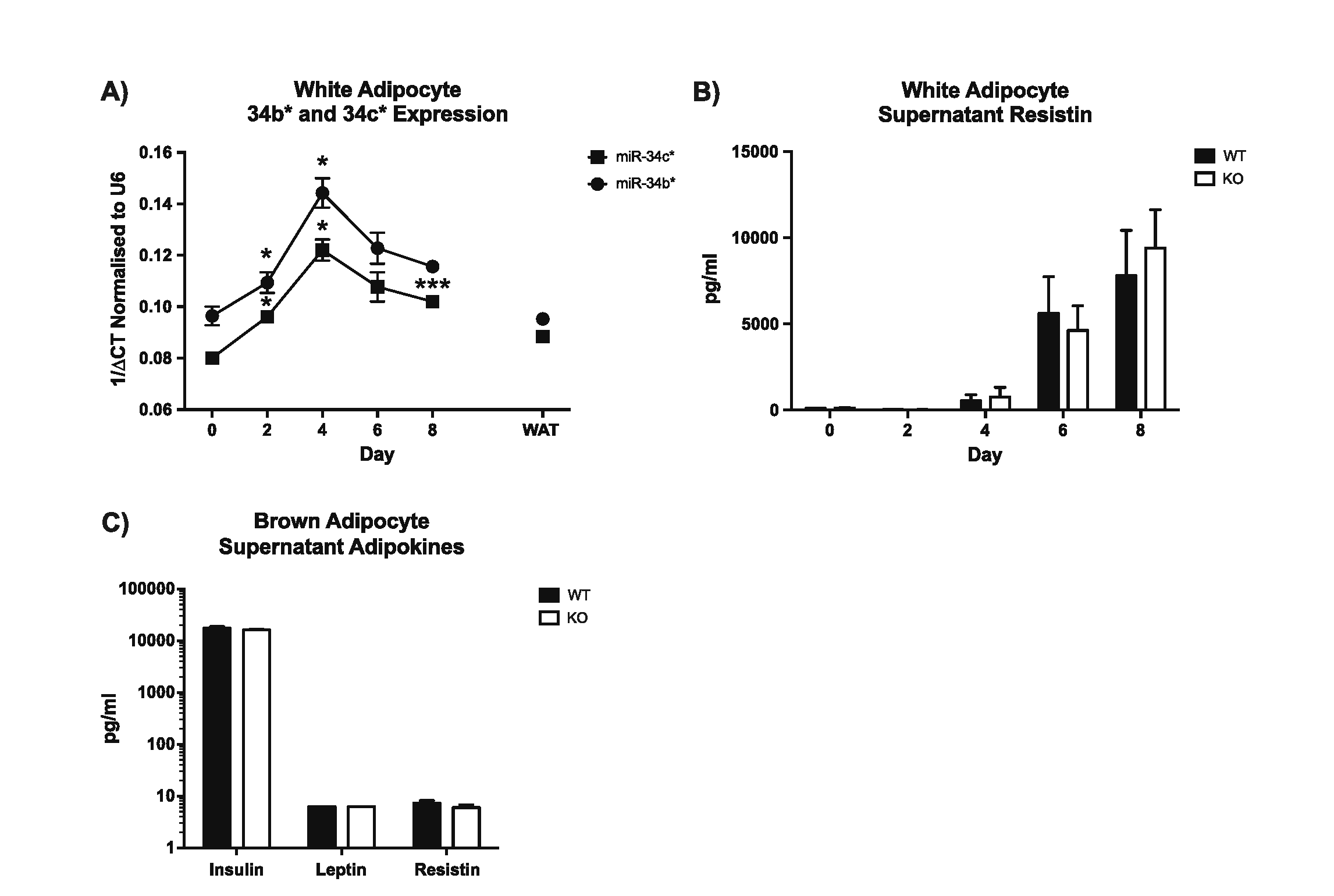


### Figure S6

(A) RT-qPCR quantification of miR-34b* and 34c* transcripts over *in vitro* differentiation of SVF white pre-adipocytes (day 0-8) from WT murine eWAT, with mature adipocyte fraction (WAT) as a positive control, normalised to RNU6B; 3 mice were pooled together for each replicate of n=3. Represented as mean 1/∆Ct values with SEM, and statistics were calculated compared to day 0. There were no detectable transcripts of mature miR-34b, miR-34a*, or miR-34c assays. (B) Adipokine Luminex data showing the concentration of resistin in the supernatant of WT and miR-34a^-/-^ (KO) *in vitro* SVF white adipocytes over differentiation (day 0-8); n=3. (C) Adipokine Luminex data showing the concentration of adipokines at day 8 of differentiation in WT and KO *in vitro* SVF brown adipocytes; n=4. Data is represented as mean values with SEM. *P<0.05, ***P<0.001 Two-way ANOVA, with Dunnett’s (A) or Bonferroni’s (B) multiple comparisons post-test, or Unpaired t test (C). Comparisons were made between WT and KO in (B-C).

###
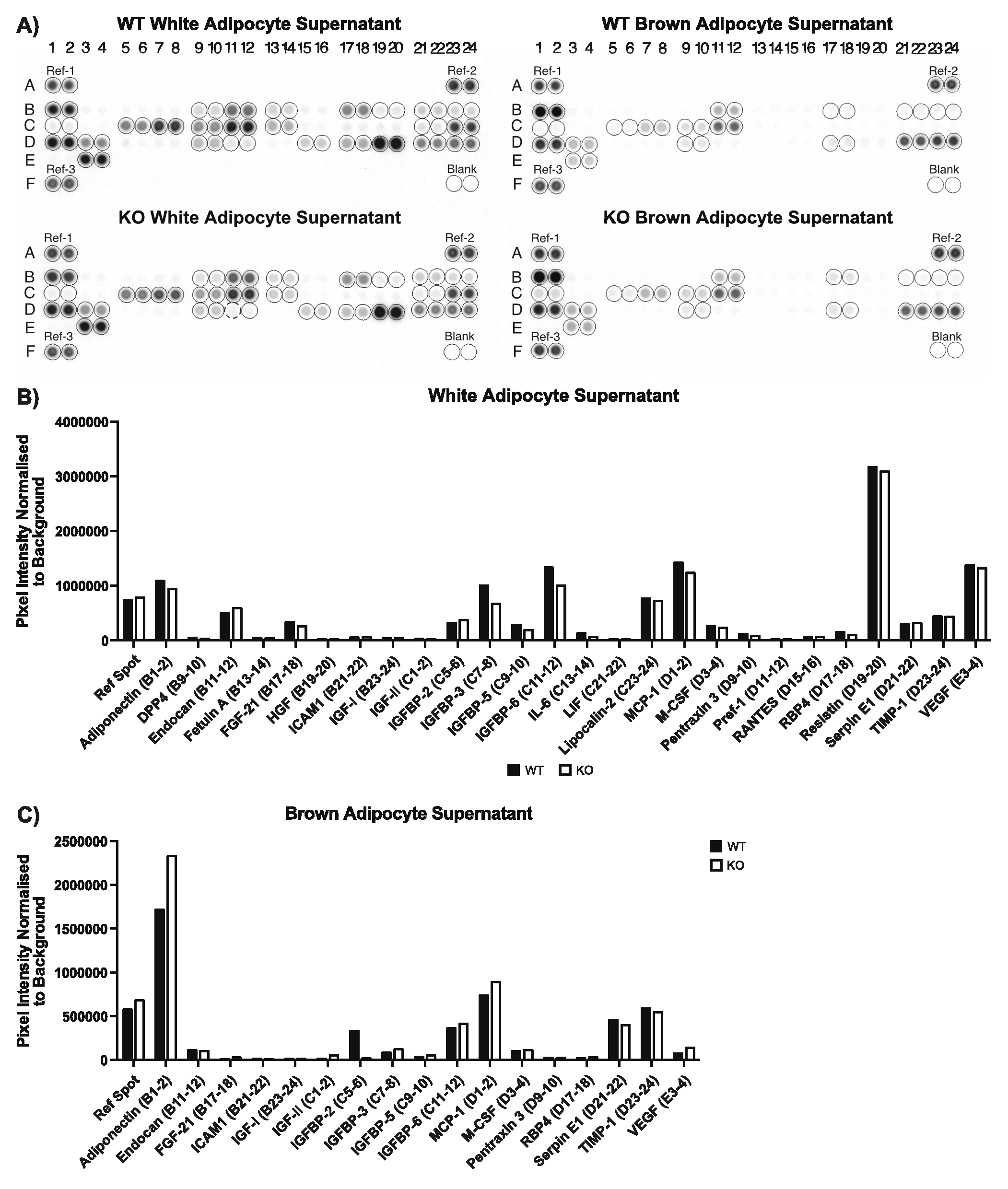


### Figure S7

Results form the Mouse Adipokine Array on supernatants from WT and miR-34a^-/-^ (KO) *in vitro* SVF white and brown adipocytes at day 8 of differentiation. The supernatants from 3 (white) or 4 (brown) separate cultures were pooled together for each group. (A) shows the array membranes, which have been quantified by densitometry for white (B) and brown (C) adipocytes, normalised to the background spots (Blank) for each membrane. Quantified data is represented as an average of the 2 spots per adipokine, or 6 spots for the positive control reference. Graph x-axis labels contain the grid number for each spot.

###
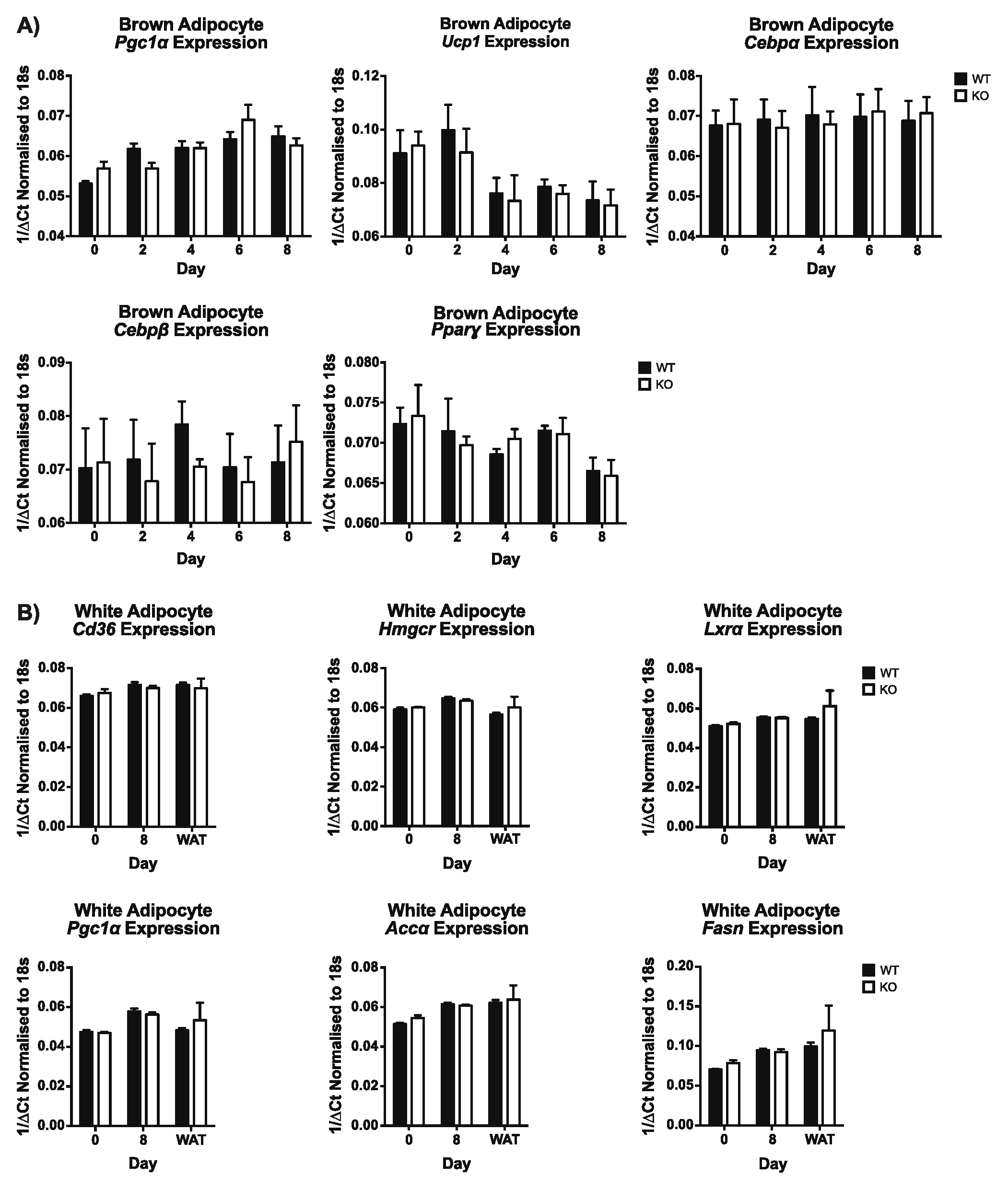


### Figure S8

RT-qPCR expression data for metabolic and differentiation genes in WT and miR-34a^-/-^ (KO) brown (A) and white (B) *in vitro* SVF adipocytes over differentiation; n=3 for white adipocytes and n=4 for brown adipocytes. Data is represented as mean 1/∆Ct values with SEM, normalised to 18s rRNA. Two-way ANOVA, with Bonferroni’s multiple comparisons post-test. Comparisons were made between WT and KO.

# References

1. Miller AM, Asquith DL, Hueber AJ, Anderson LA, Holmes WM, McKenzie AN, et al. Interleukin-33 induces protective effects in adipose tissue inflammation during obesity in mice. 2010 Sep 3;107(5):650–8.

2. Kurowska-Stolarska M, Alivernini S, Ballantine LE, Asquith DL, Millar NL, Gilchrist DS, et al. MicroRNA-155 as a proinflammatory regulator in clinical and experimental arthritis. Proceedings of the National Academy of Sciences. 2011 Jul 5;108(27):11193–8.
